# Correction to ‘MCPIP1 ribonuclease exhibits broad-spectrum antiviral effects through viral RNA binding and degradation’

**DOI:** 10.1093/nar/gkae488

**Published:** 2024-06-03

This is a correction to: Ren-Jye Lin, Hsu-Ling Chien, Shyr-Yi Lin, Bi-Lan Chang, Han-Pang Yu, Wei-Chun Tang, Yi-Ling Lin, MCPIP1 ribonuclease exhibits broad-spectrum antiviral effects through viral RNA binding and degradation, *Nucleic Acids Research*, Volume 41, Issue 5, 1 March 2013, Pages 3314–3326, https://doi.org/10.1093/nar/gkt019

In January 2024, the Editors were alerted to several issues with the supplementary Figures S2 and S5, as detailed below.

- Figure S2AB: panel A appears identical to panel B.
- Figure S2C: panel C Dox(-) and Dox(+) plates appear identical.
- Figure S5: panel C MCPIP1 negative JEV/NS1 and DAPI images appear identical to MCPIP1 C306R negative JEV/NS1 and DAPI images. Panel D MCPIP1 negative DAPI image appears identical to MCPIP1 C306R negative DAPI image.

Upon inspection, the Editors also detected an additional issue with Figure S2A:

- Figure S2A: panel MCPIP1 negative JEV/NS1 and DAPI images appear identical to panel MCPIP1 D141N negative JEV/NS1 and DAPI images, but shifted.

The authors acknowledge the duplications, have provided replacement files, and have responded as follows:

- Figure S2AB: panel A is identical to panel B.
- Figure S2A: panel MCPIP1 negative JEV/NS1 and DAPI images are identical to panel MCPIP1 D141N negative JEV/NS1 and DAPI images.

Response: In Supplementary Figure S2, we showed that Dox-induced MCPIP1, but not the enzyme-dead D141N mutant, effectively blocked viral replication, in consistence with the western blot data shown in the Fig. 2A and 2B of the main text. The panel B in Figure S2 (anti-dengue virus result) was inadvertently duplicated from panel A (anti-Japanese encephalitis virus result). In addition, panel MCPIP1 negative JEV/NS1 and DAPI images appear identical to panel MCPIP1 D141N negative JEV/NS1 and DAPI images. We have found the right immunofluorescence photos and would like to correct panels A and B as shown in the new Figure S2 in the Supplementary Data. The original images for both panels are also in the Supplementary Data.

- Figure S2C: panel C Dox(-) and Dox(+) plates are identical.

Response: Panel C in Figure S2 showed the representative JEV plaque forming images with the calculated virus titer results shown in Fig. 2C of the main text. The plaque images of vector control with or without Dox treatment have been inadvertently duplicated. Without Dox treatment, the indicated proteins were not expressed. To avoid confusion, we would like to remove the Dox (-) panel and show only the panel Dox (+). The new Figure S2 is in the Supplementary Data.

- Figure S5: Panel C MCPIP1 negative JEV/NS1 and DAPI images are identical to MCPIP1 C306R negative JEV/NS1 and DAPI images. Panel D MCPIP1 negative DAPI image is identical to MCPIP1 C306R negative DAPI image.

Response: All immunofluorescent photos, save one, in Supplementary figure S5 panels C and D have been misplaced. We would like to replace panel C with experiments done on 30-Jun-2010. We would like to replace panel D with experiments done on 01-Mar-2010. An entirely new Figure S5 is in the Supplementary Data. The original images are also in the Supplementary Data.

This correction does not affect the results, discussion and conclusions presented in the article. These details have been corrected only in this correction notice to preserve the published version of record.

## Supplementary data

Supplementary Data are available at NAR Online.

## Supplementary Material

gkae488_Supplemental_Files

