# Supplementary figures and images for: Correction to ‘MCPIP1 ribonuclease exhibits broad-spectrum antiviral effects through viral RNA binding and degradation’
Source: Nucleic Acids Res. 2024 Jun 3;52(12):7398. doi: 10.1093/nar/gkae488 (PMC11229314; doi:10.1093/nar/gkae488)

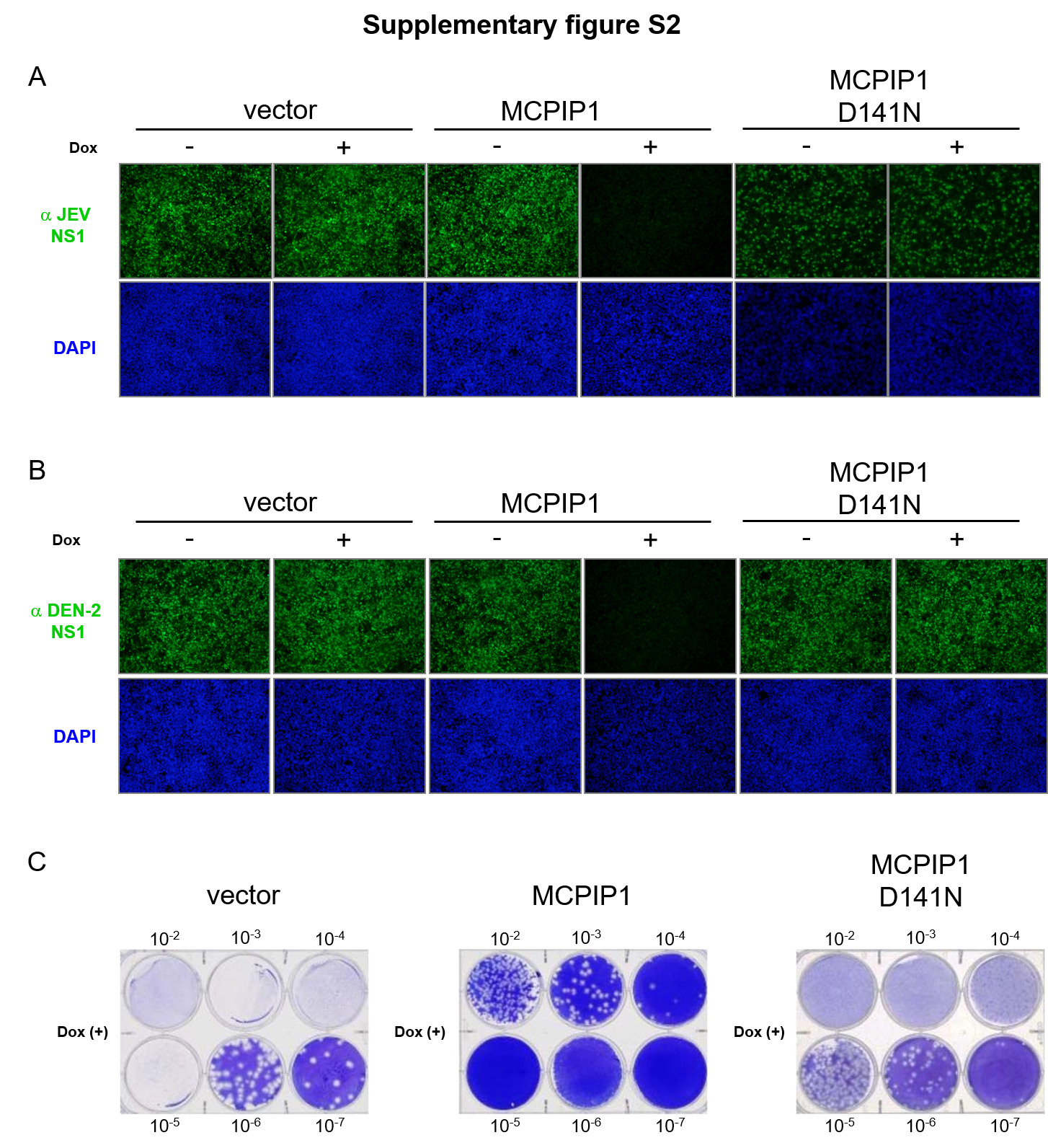

Supplement: gkae488_Supplemental_Files [file gkae488_supplemental_files.zip › Supplementary_figure_S2.tif]

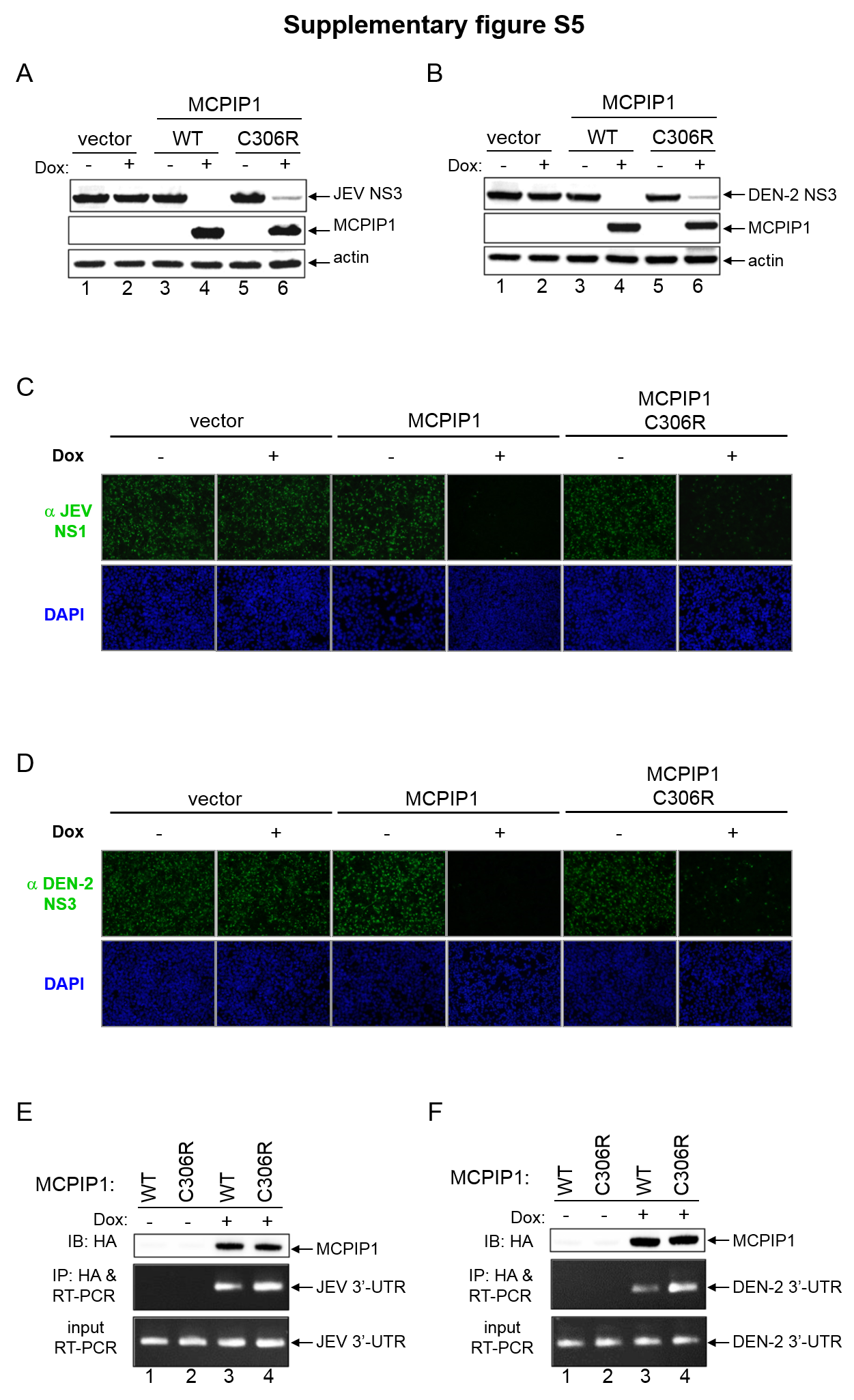

Supplement: gkae488_Supplemental_Files [file gkae488_supplemental_files.zip › Supplementary_figure_S5.tiff]
